# Supplementary figures and images for: Relevance of CYP2D6 Gene Variants in Population Genetic Differentiation
Source: Pharmaceutics. 2022 Nov 16;14(11):2481. doi: 10.3390/pharmaceutics14112481 (PMC9694252; doi:10.3390/pharmaceutics14112481)

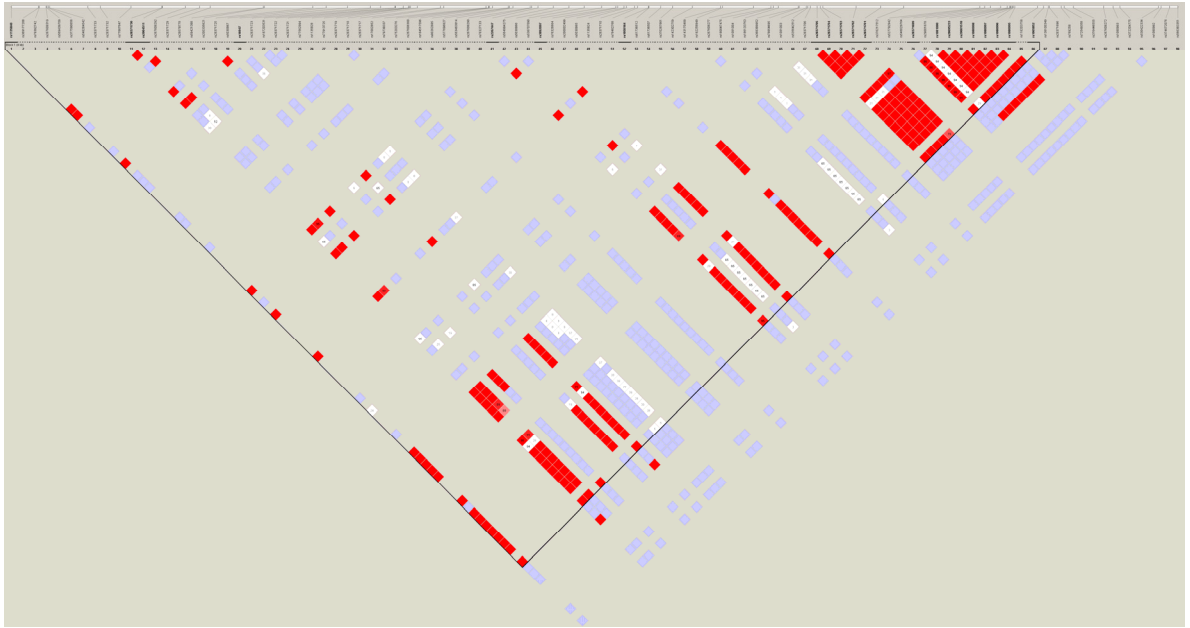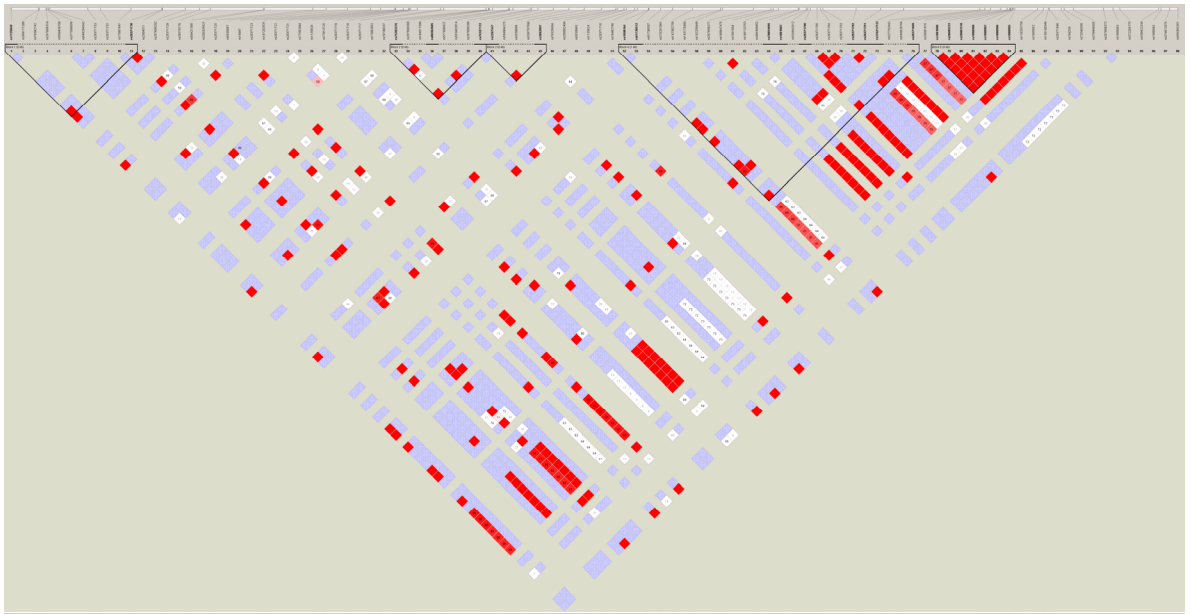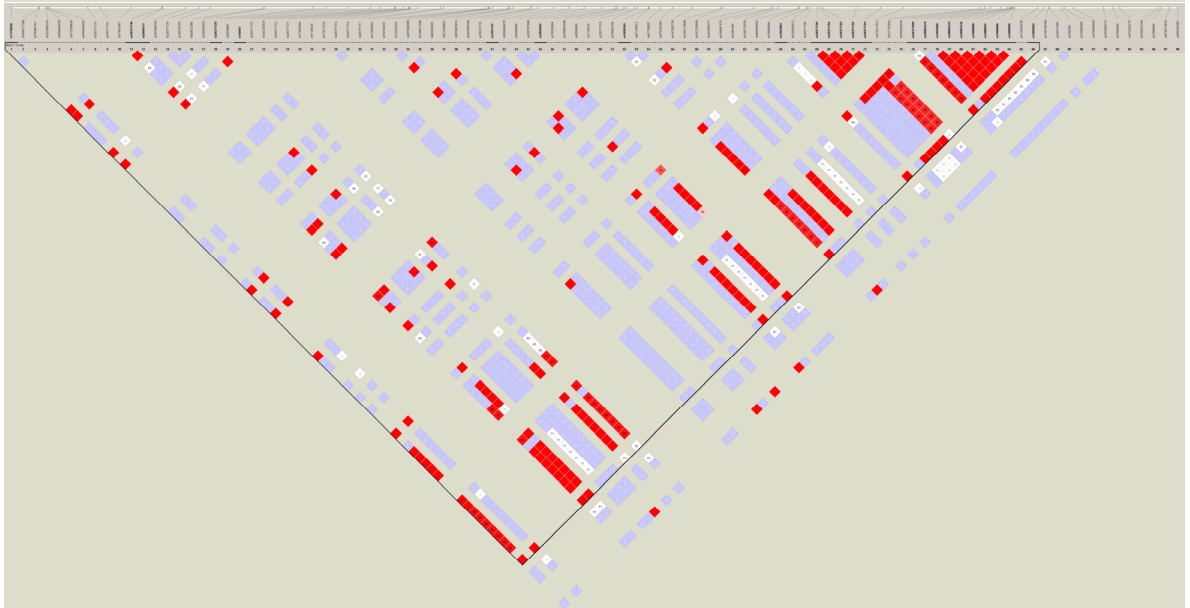

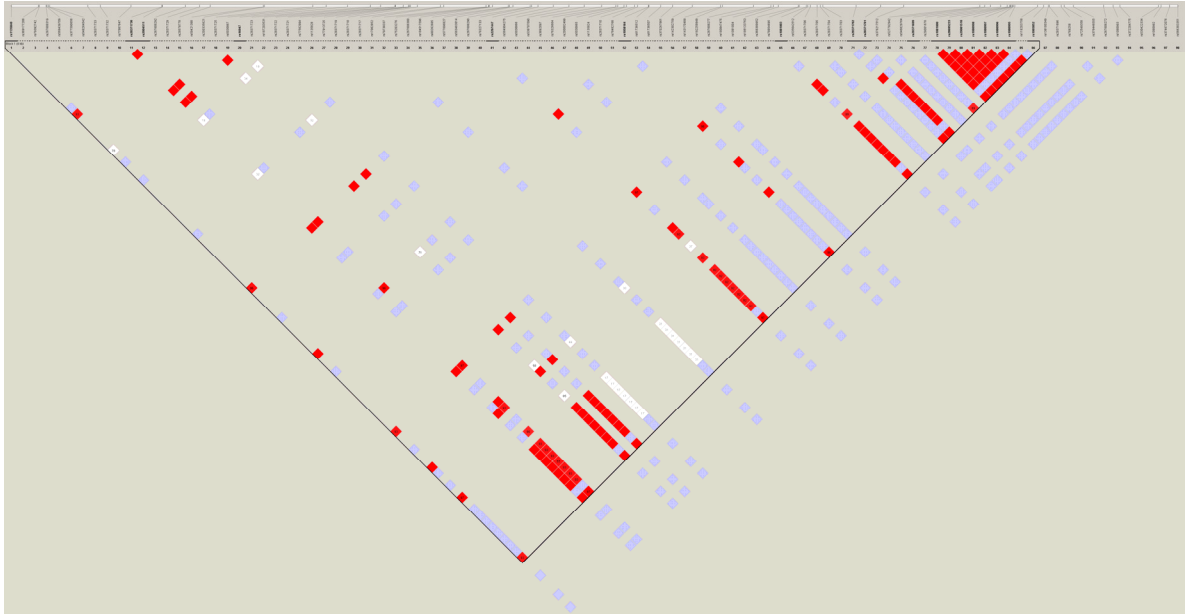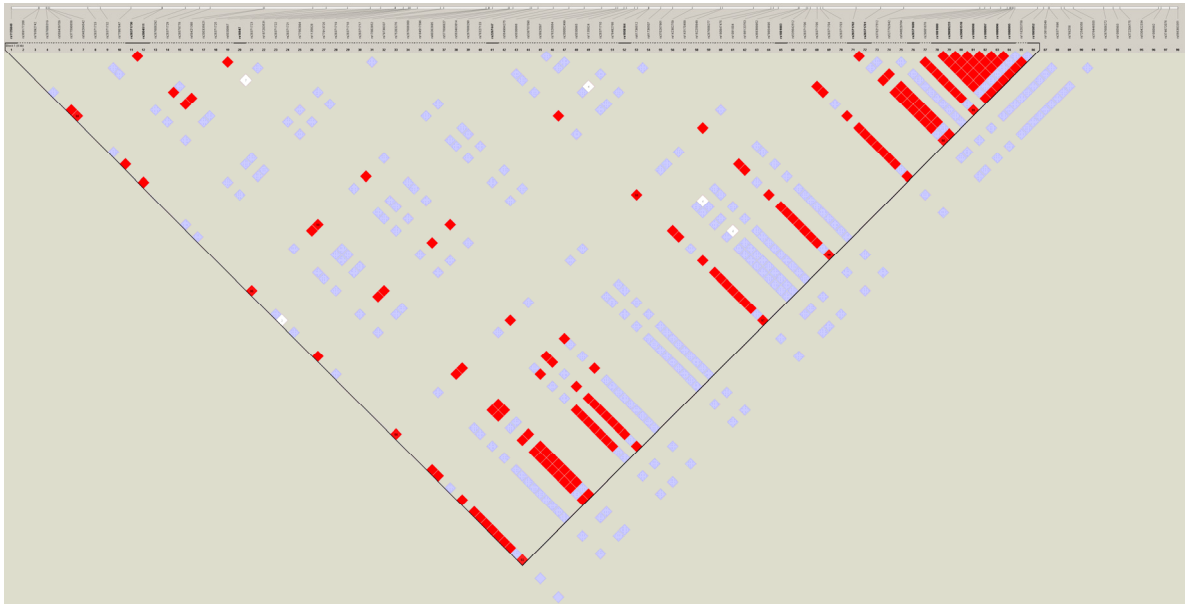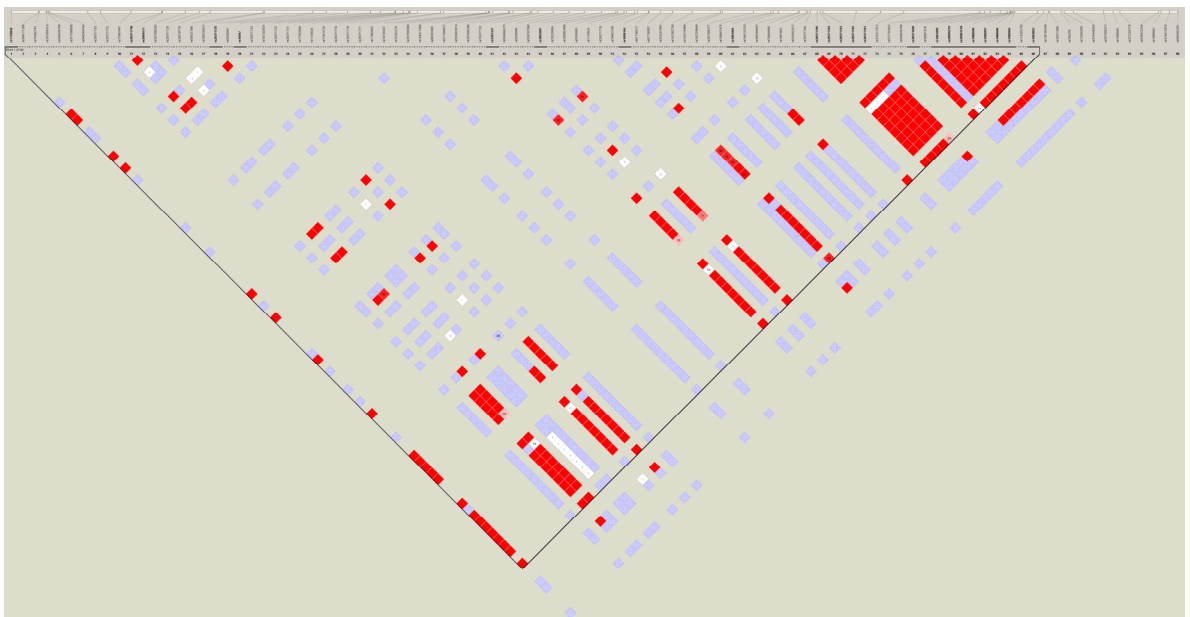

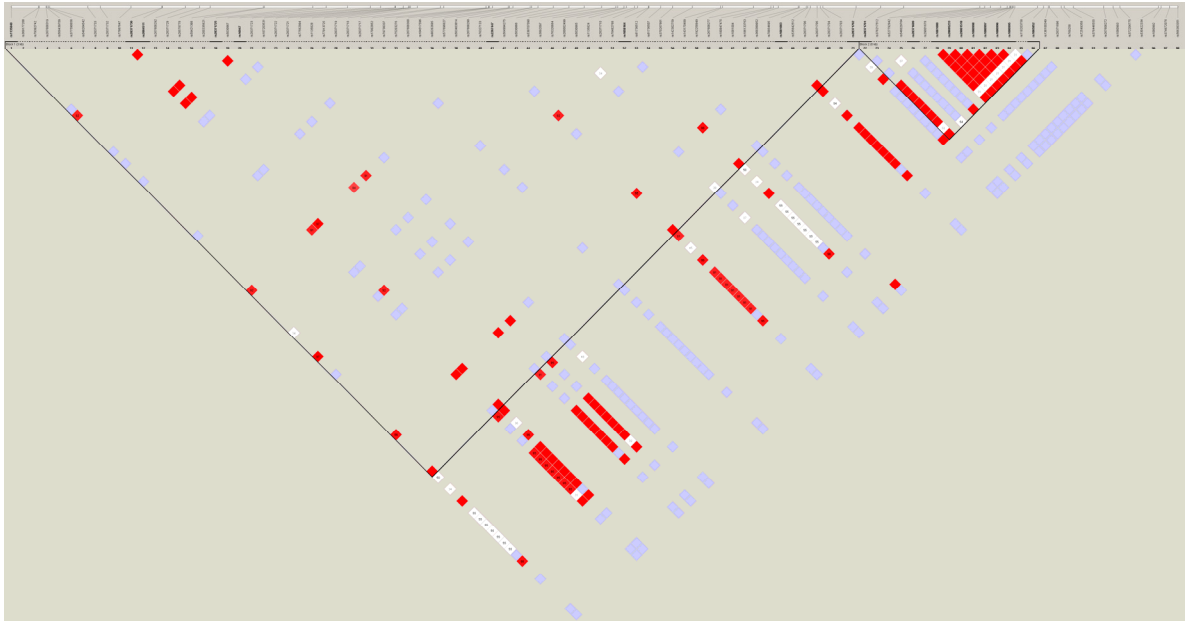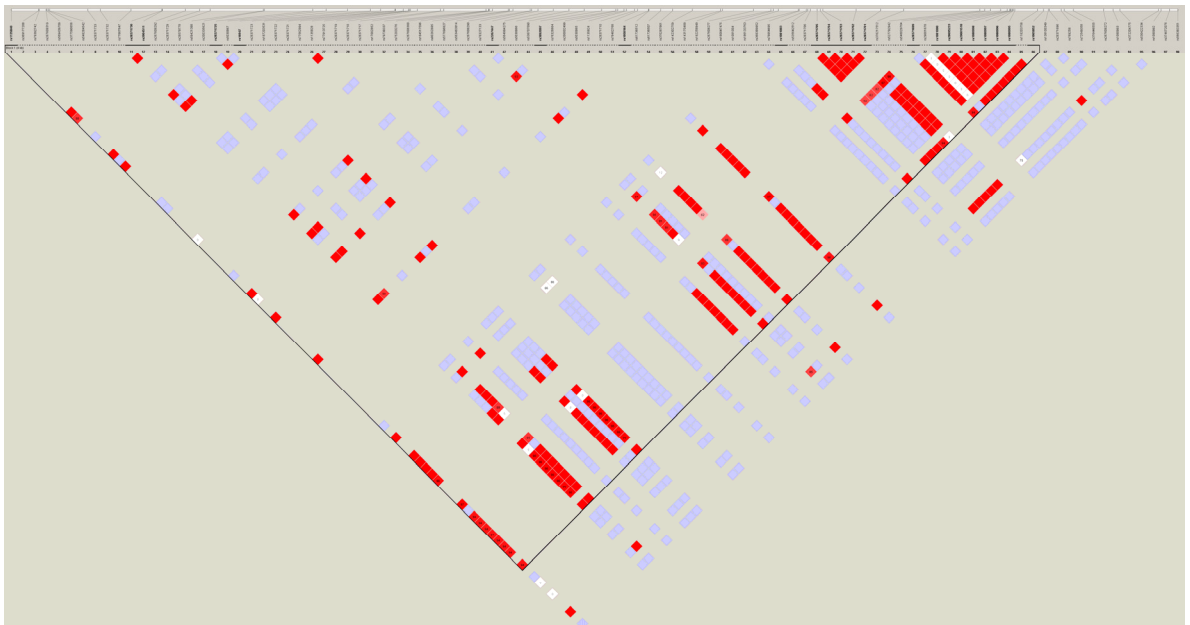

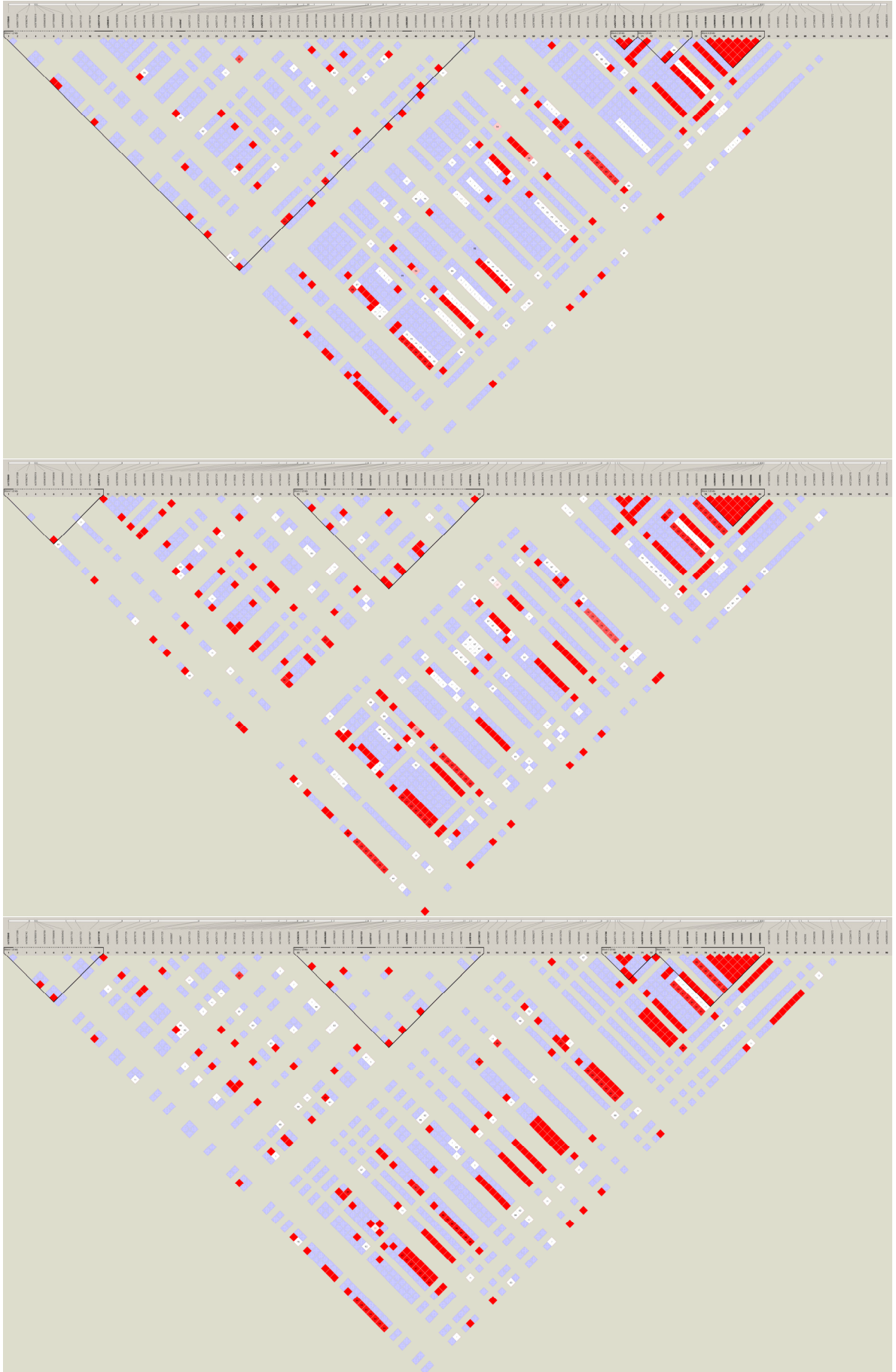

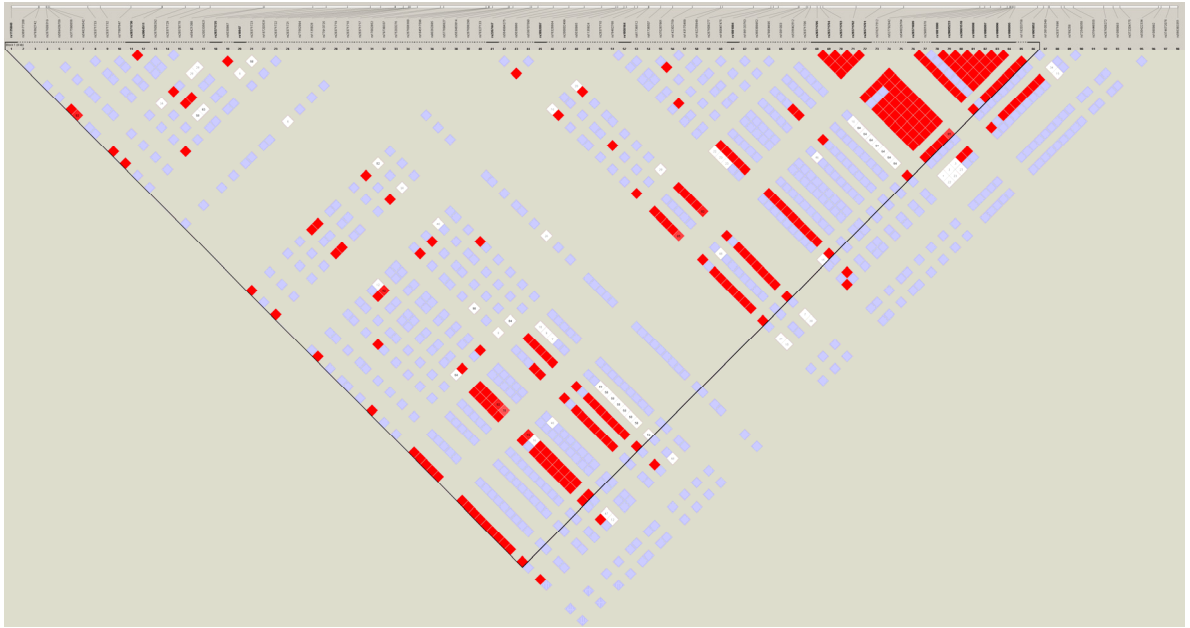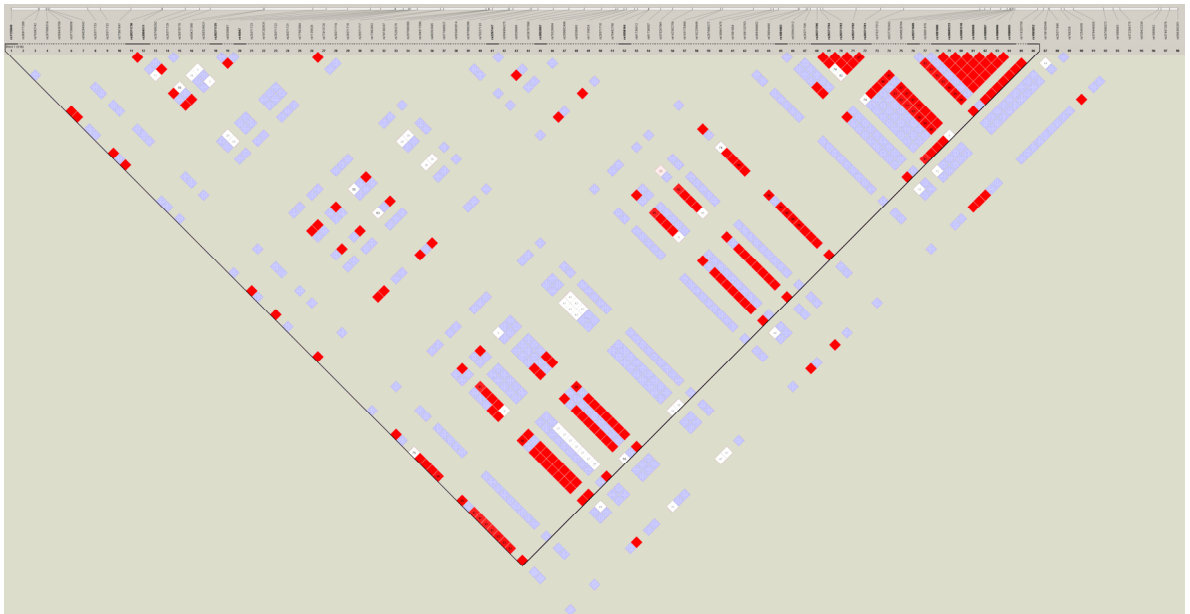

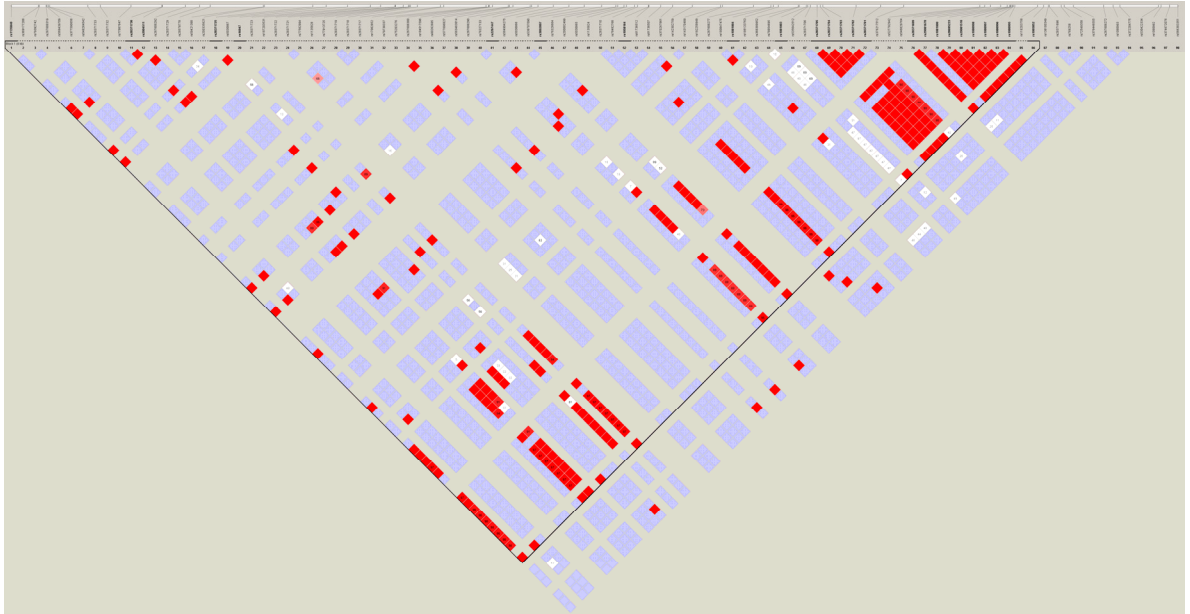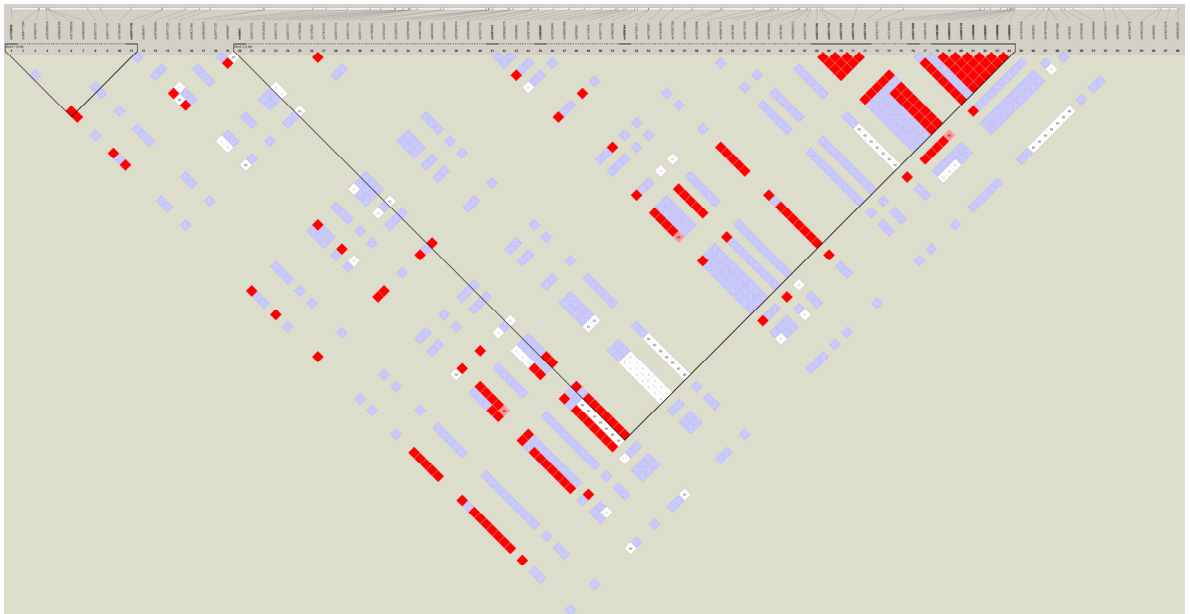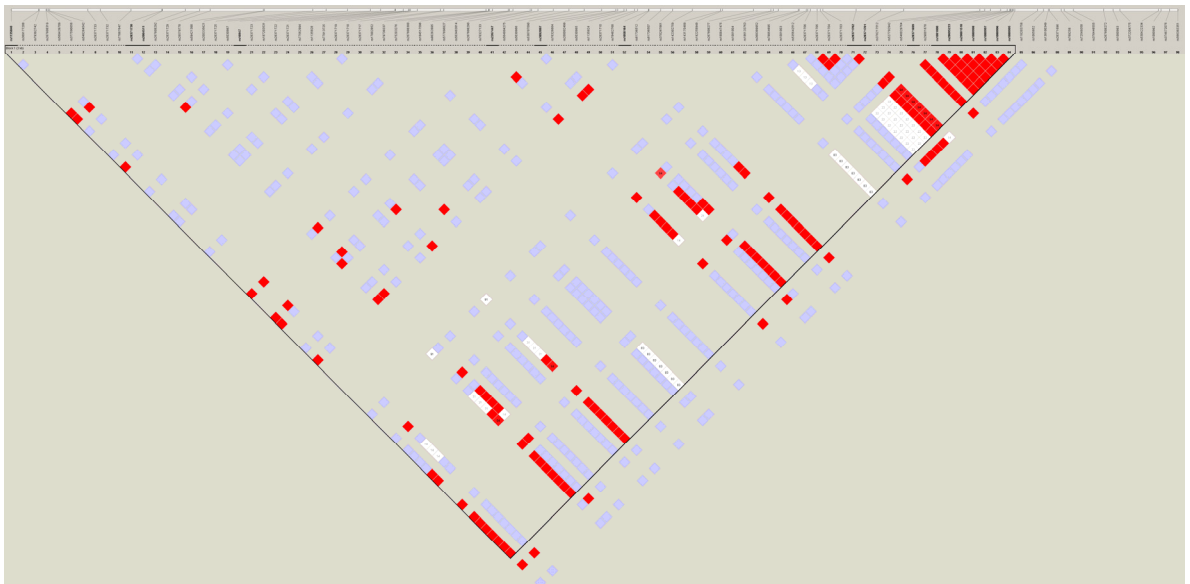

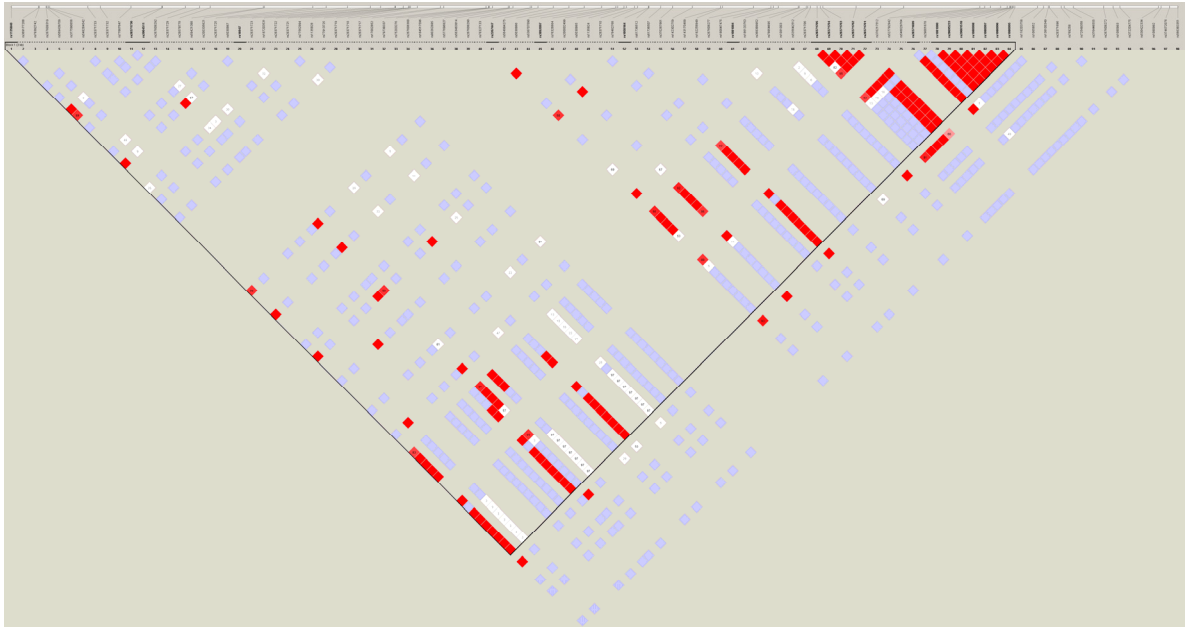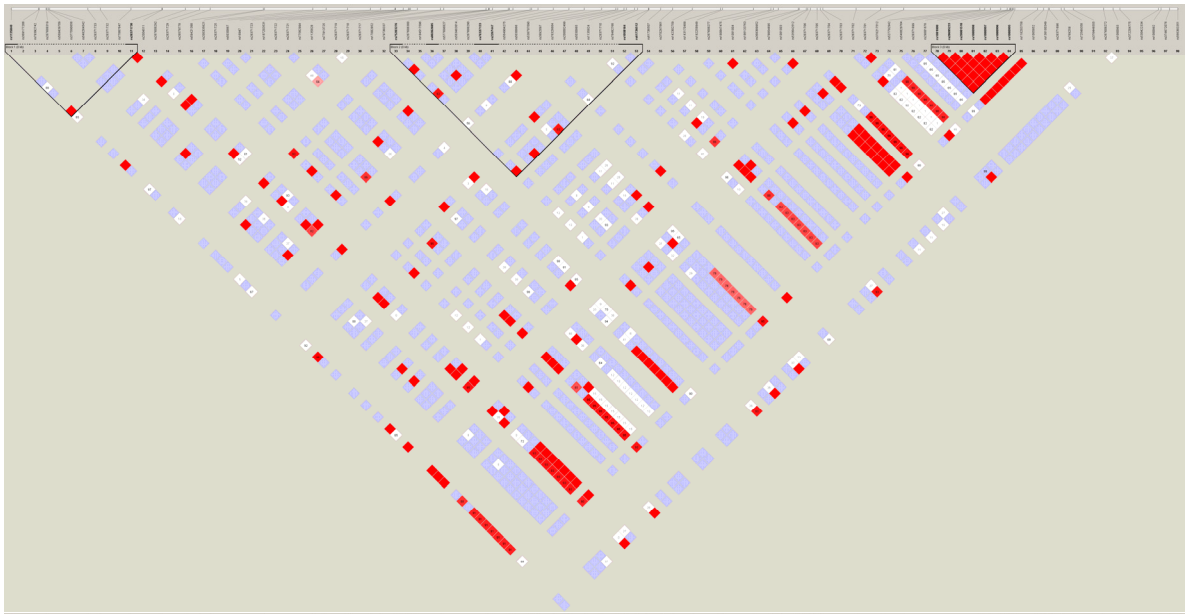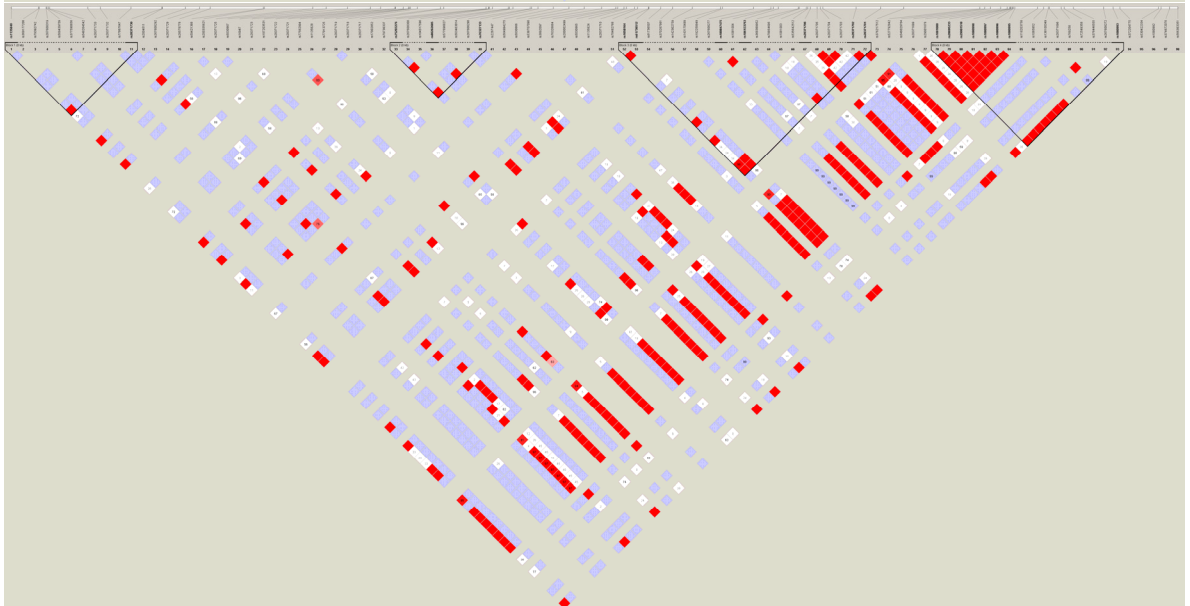

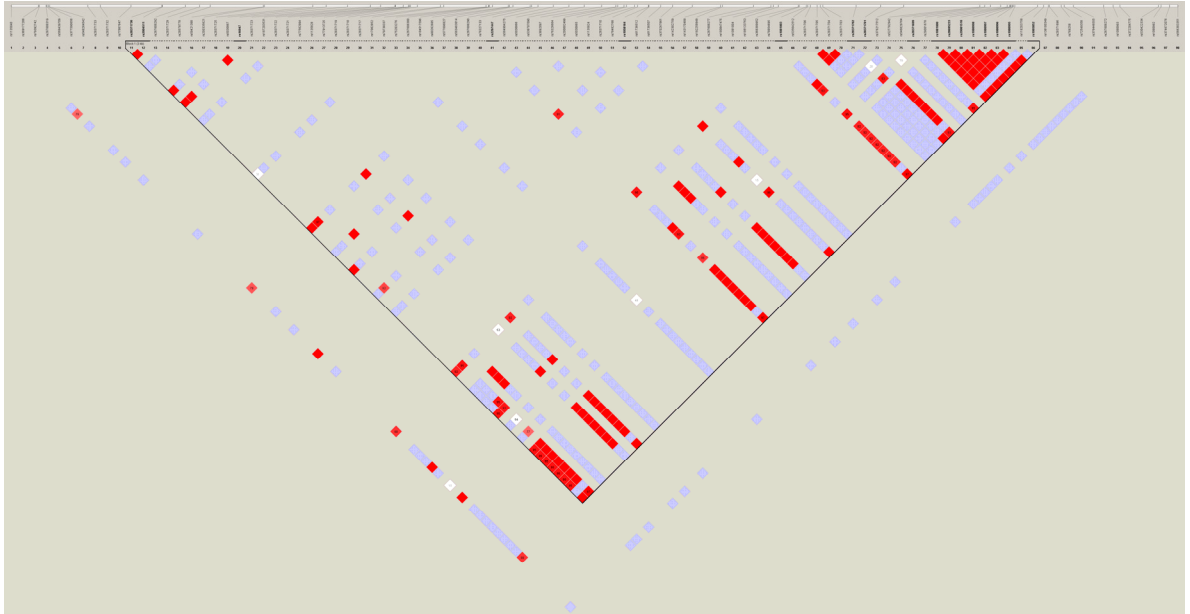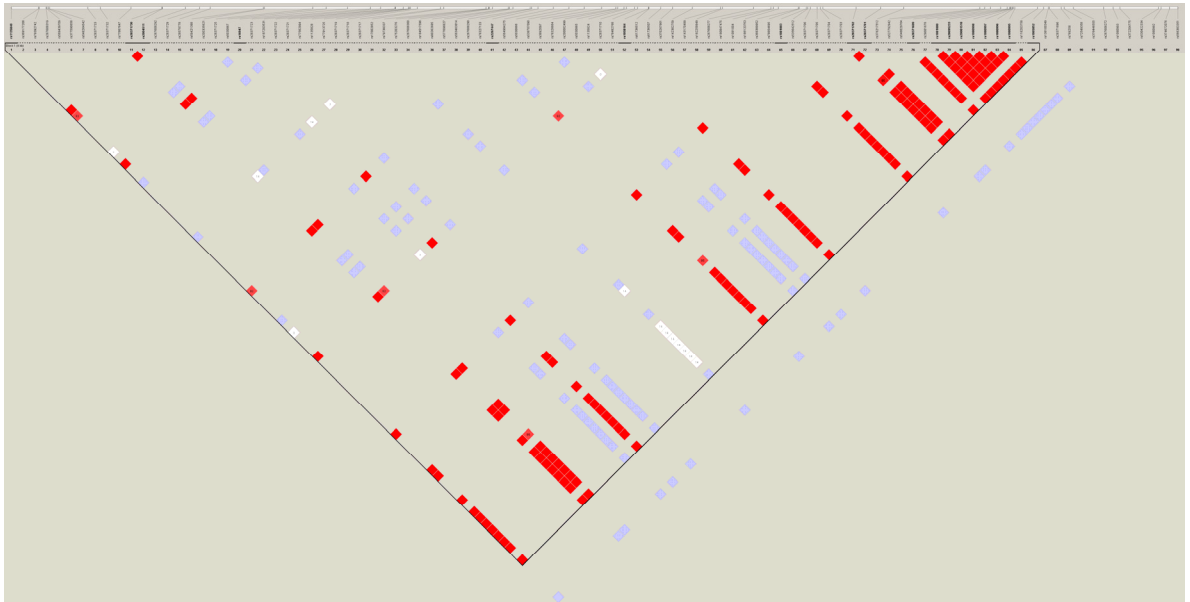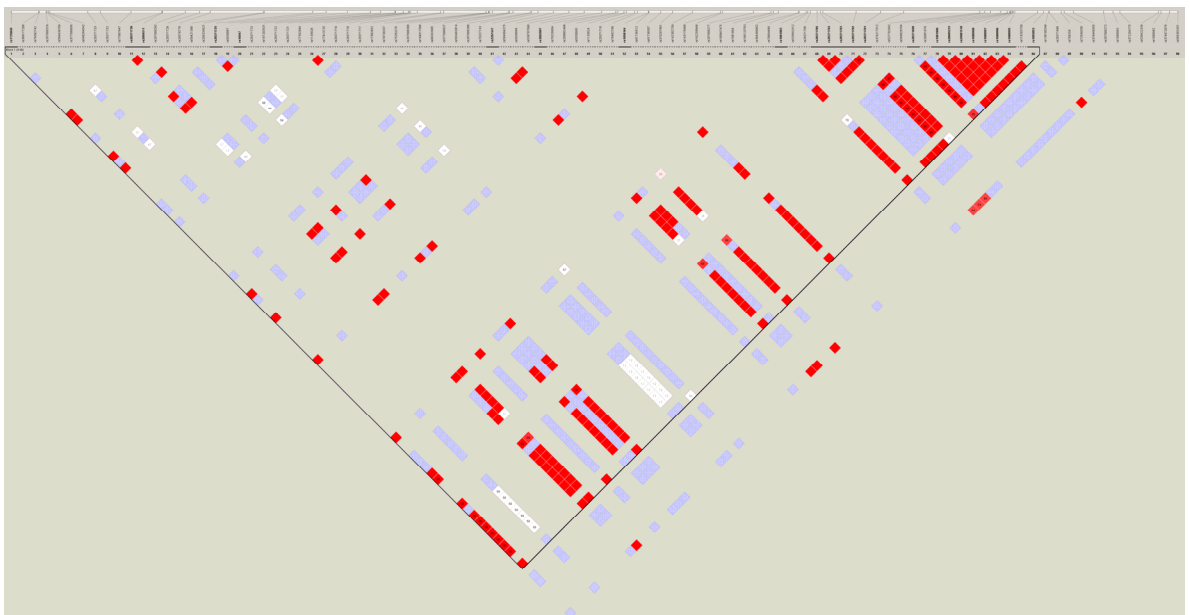

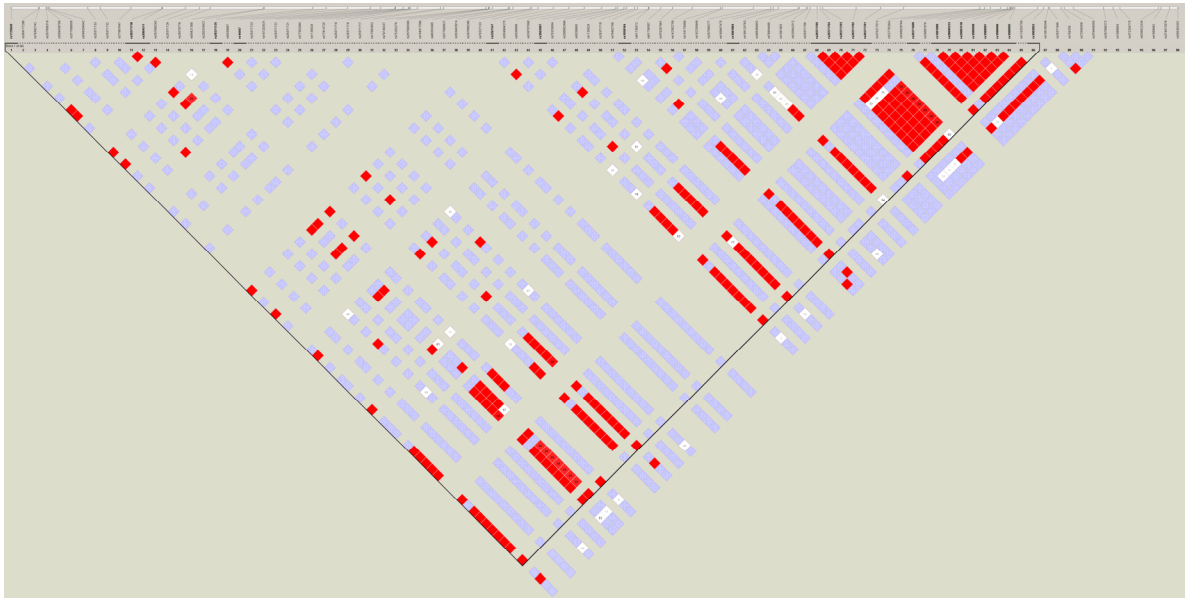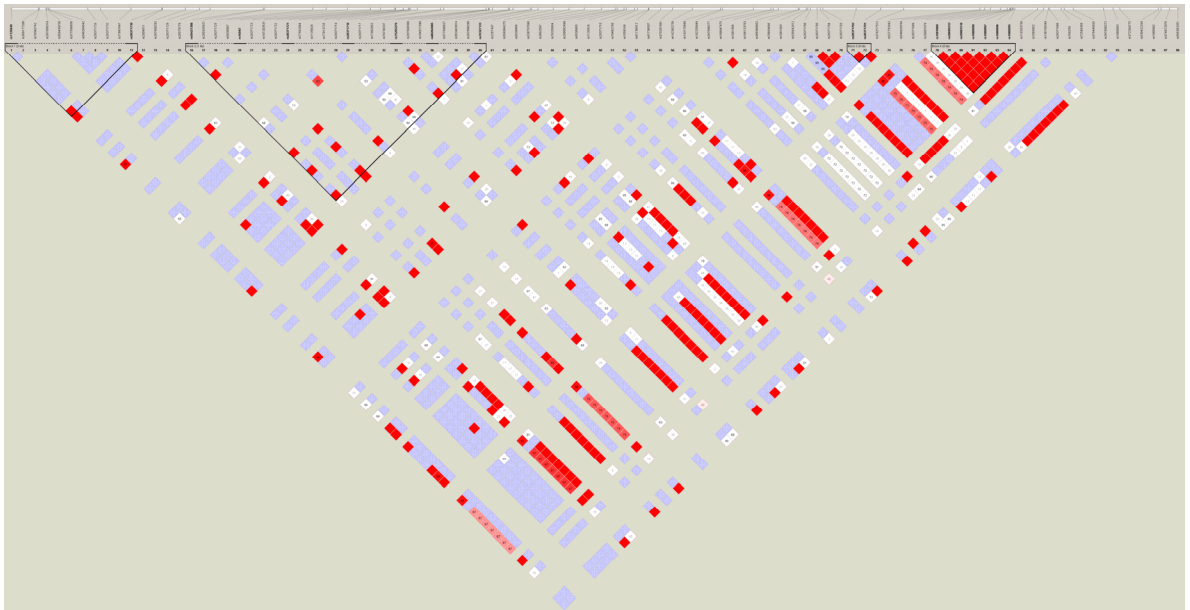

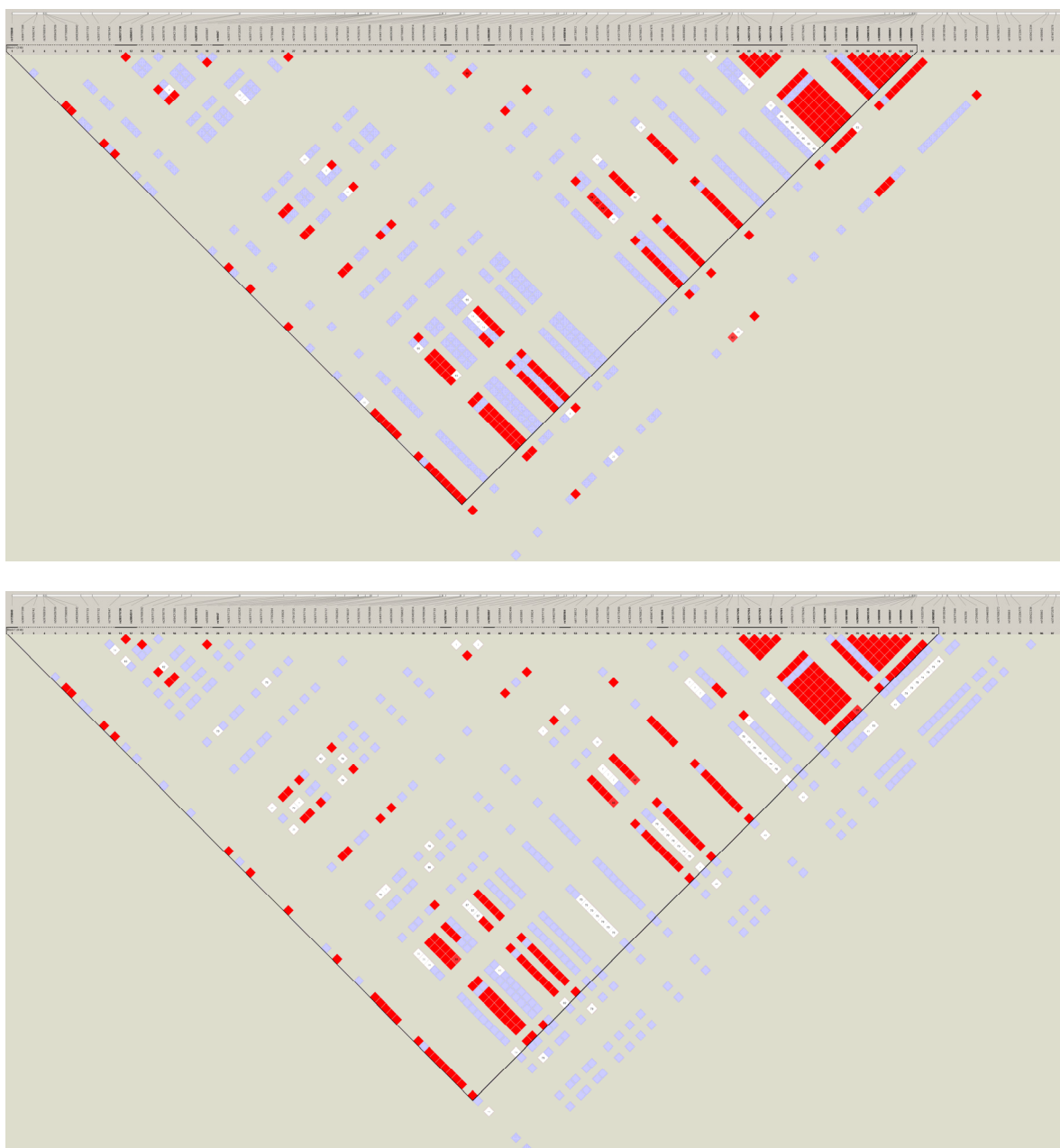

**Figure S1:** Haplotype blocks for 26 world populations.

Supplement: Supplementary file 1 [file pharmaceutics-14-02481-s001.zip › Figure S1.pdf]
